# Antitoxic Action of Charcoal

**Published:** 1914-11

**Authors:** 


**Antitoxic Action of Charcoal.** Wielchowski and Adler, Ther. der Gegenwart, 1914, No. 5, confirm the time-honored view of the absorptive power of charcoal. Poisons administered with charcoal become inert — with obvious qualifications — and methylene blue is fixed so that it does not appear in the urine.

